# Elevated plasma levels of the appetite-stimulator ACBP/DBI in fasting and obese subjects

**DOI:** 10.15698/cst2021.07.252

**Published:** 2021-06-28

**Authors:** Sijing Li, Adrien Joseph, Isabelle Martins, Guido Kroemer

**Affiliations:** 1 Centre de Recherche des Cordeliers, Equipe labellisée par la Ligue contre le cancer, Inserm U1138, Université de Paris, Sorbonne Université, Paris, France.; 2 Metabolomics and Cell Biology Platforms, Institut Gustave Roussy, Villejuif, France.; 3 Faculté de Médecine, Université de Paris Saclay, Kremlin Bicêtre, France.; 4 Pôle de Biologie, Hôpital Européen Georges Pompidou, AP-HP, Paris, France.; 5 Suzhou Institute for Systems Medicine, Chinese Academy of Medical Sciences, Suzhou, China.; 6 Karolinska Institute, Department of Women's and Children's Health, Karolinska University Hospital, Stockholm, Sweden.; # SL and AJ equally contributed to this paper.

**Keywords:** metabolism, obesity, diazepam binding protein, appetite, starvation, autophagy

## Abstract

Eukaryotic cells release the phylogenetically ancient protein acyl coenzyme A binding protein (ACBP, which in humans is encoded by the gene DBI, diazepam binding inhibitor) upon nutrient deprivation. Accordingly, mice that are starved for one to two days and humans that undergo voluntary fasting for one to three weeks manifest an increase in the plasma concentration of ACBP/DBI. Paradoxically, ACBP/DBI levels also increase in obese mice and humans. Since ACBP/DBI stimulates appetite, this latter finding may explain why obesity constitutes a self-perpetuating state. Here, we present a theoretical framework to embed these findings in the mechanisms of weight control, as well as a bioinformatics analysis showing that, irrespective of the human cell or tissue type, one single isoform of ACBP/DBI (ACBP1) is preponderant (~90% of all DBI transcripts, with the sole exception of the testis, where it is ~70%). Based on our knowledge, we conclude that ACBP1 is subjected to a biphasic transcriptional and post-transcriptional regulation, explaining why obesity and fasting both are associated with increased circulating ACBP1 protein levels.

## INTRODUCTION

Acyl coenzyme A binding protein (ACBP), which in humans is encoded by the gene *DBI* (diazepam binding inhibitor), is a phylogenetically ancient protein that is ubiquitously expressed by all nucleated human cell types [1–3]. Reflecting its double name, ACBP/DBI has two distinct functions, namely as an intracellular contributor to fatty acid metabolism and as an extracellular mediator that binds to the γ-aminobutyric acid A receptor (GABAAR), competing for benzodiazepine binding (which explains the name DBI) and acting as an inverse agonist [4–6]. Recently, ACBP/DBI has been suggested to have a major obesogenic effect, based on the observation that intraperitoneal injection of recombinant ACBP/DBI protein into mice stimulates food intake and lipo-anabolic reactions, while, conversely, neutralization of ACBP/DBI by intraperitoneal injection of suitable antibodies reduces appetite and stimulates a lipo-catabolic metabolism, thereby preventing high-fat diet-induced obesity [7, 8].

Based on the likely pathophysiological relevance of ACBP/DBI in appetite control [9], we investigated which isoforms of the protein are expressed in human tissues. Moreover, we screened transcription databases and reviewed the literature to understand why two apparently antinomic states, fasting and obesity can both result in an elevation of circulating ACBP/DBI protein levels.

**Figure 1:**
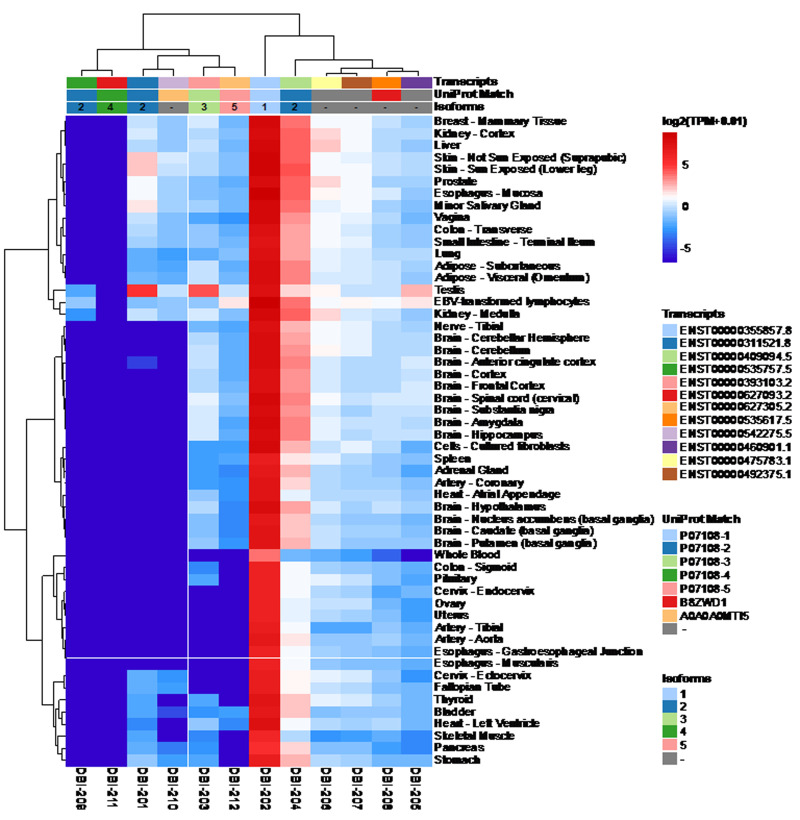
FIGURE 1: The expression of DBI isoforms in human tissues. The data were obtained from GTExPortal database (https://www.gtexportal.org/home/) on April, 7th 2021. Expression values are shown in TPM (Transcripts per million, binary logarithmic scale).

## ACBP/DBI ISOFORMS IN THE HUMAN TRANSCRIPTOME

**Figure 2:**
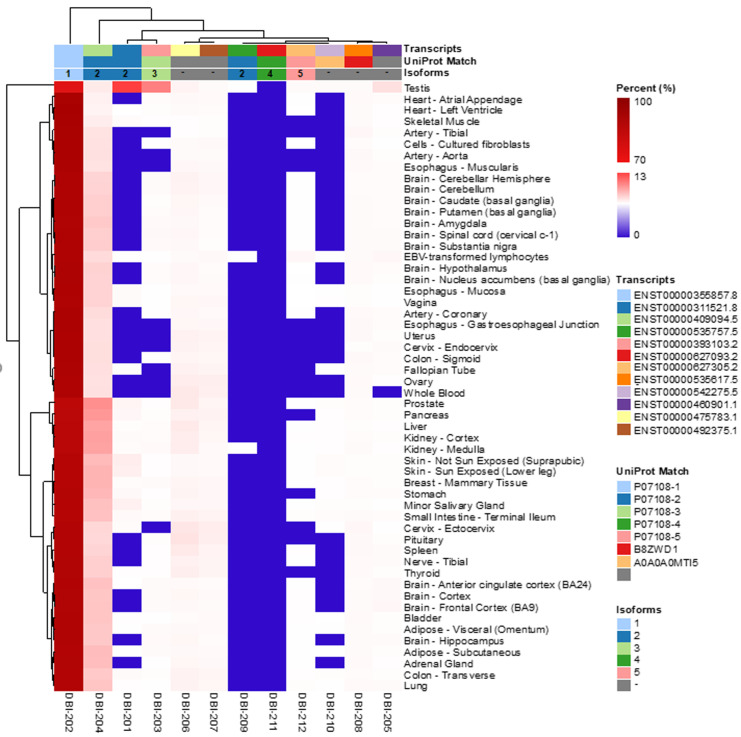
FIGURE 2: The proportion of DBI isoforms in human tissues. The data were obtained from GTExPortal database (https://www.gtexportal.org/home/) on April, 7th 2021. Expression values are shown in percentages of the different isoforms in the corresponding tissue.

According to the Uniprot website (https://www.uniprot.org/), ACBP/DBI may give rise to six different ACBP isoforms (numbered as ACBP1 to ACBP6) that have been detected by mRNA sequencing, as well as two additional isoforms (ACBP7 and ACBP8) that are predicted by computation based on the Uniprot dataset (https://www.uniprot.org/; **Table 1**). We subsequently identified the corresponding transcripts, whenever possible, as well as transcripts of the ACBP/DBI gene that, according to theoretical predictions, are unlikely to code for peptides/proteins. Of note, for the ACBP2 isoform, several distinct transcripts (which only differ in non-coding regions) have been identified by deep sequencing. We then interrogated the GTExPortal (https://www.gtexportal.org/home/) to define the relative abundance of each transcript in distinct human tissues. The transcript encoding the ACBP1 isoform was uniformly expressed at high levels (with the sole exception of whole blood cells), in accord with the observation that the ACBP protein (detected by immunohistochemistry) is present in all human tissues [9]. In all tissues, ACBP2 is the second-most expressed isoform. Of note, in testis ACBP2 is encoded by a different transcript (ENST00000492375.1) than in all other organs (where transcript ENST000003111521.8 prevails). Moreover, testis is the sole organ that expresses significant levels of ACBP3. All other ACBP isoforms (ACBP4, ACBP5, ACBP6) were scarcely expressed (**Fig. 1**). The difference in the abundance of different ACBP isoforms becomes particularly clear when the expression level of all isoforms is set to 100%. ACBP1 clearly represents the dominant ACBP/DBI isoform (with values close to or higher than 90%) with the sole exception of testis in which ACBP1 represents only 71% (**Fig. 2**). This may reflect the fact that the testicular transcriptome/proteome is unique due to expression of genes that are repressed in adult tissues, but transactivated in germline cells [10, 11]. In conclusion, ACBP1 is the quantitatively most important ACBP/DBI isoform expressed in human tissues.

**TABEL 1.** Transcripts of the DBI gene and isoforms of the DBI protein.

**Isoform**	**Name**	**Transcript ID**	**bp**	**Translation ID**	**CCDS**	**UniProt**	**Exon**	**Amino acid sequences**
1 (ACBP-1a)	DBI-202	ENST00000355857.8	564	ENSP00000348116.3	CCDS42740	P07108-1	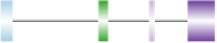 ENSE00003772551/ENSE00003674746 ENSE00003592668/ENSE00002317965 1 (119366977-119367060 _84bp) 2 (119368188-119368305 _118bp) 3 (119370740-119370802 _63bp) 4 (119372245-119372543 _299bp)	MSQAEFEKAAEEVRHLKTKPSDEEMLFIYGHYKQATVGDINTERPGMLDFTGKAKWDAWNELKGTSKEDAMKAYINKVEELKKKYGI (87 aa)
2 (ACBP-1b)	DBI-201	ENST00000311521.8	714	ENSP00000311117.4	CCDS2126	P07108-2	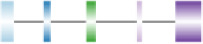 ENSE00001425667/ENSE00001140850 ENSE00003674746/ENSE00003592668 ENSE00001202561 1 (119367070-119367214 _145bp) 2 (119367559-119367644 _86bp) 3 (119368188-119368305 _118bp) 4 (119370740-119370802 _63bp) 5 (119372245-119372546 _302bp)	MWGDLWLLPPASANPGTGTEAEFEKAAEEVRHLKTKPSDEEMLFIYGHYKQATVGDINTERPGMLDFTGKAKWDAWNELKGTSKEDAMKAYINKVEELKKKYGI (104 aa)
2 (ACBP-1b)	DBI-204	ENST00000409094.5	576	ENSP00000386486.1	CCDS2126	P07108-2	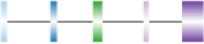 ENSE00001582563/ENSE00001140850 ENSE00003674746/ENSE00003592668 ENSE00001585219 1 (119366989-119367060 _72bp) 2 (119367559-119367644 _86bp) 3 (119368188-119368305 _118bp) 4 (119370740-119370802 _63bp) 5 (119372245-119372481 _237bp)	MWGDLWLLPPASANPGTGTEAEFEKAAEEVRHLKTKPSDEEMLFIYGHYKQATVGDINTERPGMLDFTGKAKWDAWNELKGTSKEDAMKAYINKVEELKKKYGI (104 aa)
2 (ACBP-1b)	DBI-209	ENST00000535757.5	740	ENSP00000439012.1	CCDS2126	P07108-2	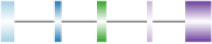 ENSE00002250725/ENSE00001140850 ENSE00003674746/ENSE00003592668 ENSE00001931282 1 (119366924-119367090 _167bp) 2 (119367559-119367644 _86bp) 3 (119368188-119368305 _118bp) 4 (119370740-119370802 _63bp) 5 (119372245-119372550 _306bp)	MWGDLWLLPPASANPGTGTEAEFEKAAEEVRHLKTKPSDEEMLFIYGHYKQATVGDINTERPGMLDFTGKAKWDAWNELKGTSKEDAMKAYINKVEELKKKYGI (104 aa)
3 (ACBP-1c)	DBI-203	ENST00000393103.2	599	ENSP00000376815.2	CCDS42741	P07108-3	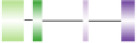 ENSE00001514188/ENSE00003674746 ENSE00003592668/ENSE00001852476 1 (119367677-119367931 _255bp) 2 (119368188-119368305 _118bp) 3 (119370740-119370802 _63bp) 4 (119372245-119372407 _163bp)	MPAFAEFEKAAEEVRHLKTKPSDEEMLFIYGHYKQATVGDINTERPGMLDFTGKAKWDAWNELKGTSKEDAMKAYINKVEELKKKYGI (88 aa)
4 (ACBP-1a1-g)	DBI-211	ENST00000627093.2	554	ENSP00000486281.1	CCDS54390	P07108-4	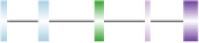 ENSE00003772551/ENSE00003773620 ENSE00003674746/ENSE00003592668 ENSE00001852476 1 (119366977-119367060 _84bp) 2 (119367403-119367528 _126bp) 3 (119368188-119368305 _118bp) 4 (119370740-119370802 _63bp) 5 (119372245-119372407 _163bp)	MSQHRAGRRGGVGKRGVRGRELGGQGKYGAGCSECGTRRIAARGEAEFEKAAEEVRHLKTKPSDEEMLFIYGHYKQATVGDINTERPGMLDFTGKAKWDAWNELKGTSKEDAMKAYINKVEELKKKYGI (129 aa)
5 (ACBP-1g)	DBI-212	ENST00000627305.2	620	ENSP00000486361.1	CCDS54391	P07108-5	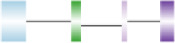 ENSE00003768948/ENSE00003674746 ENSE00003592668/ENSE00001852476 1 (119367253-119367528 _276bp) 2 (119368188-119368305 _118bp) 3 (119370740-119370802 _63bp) 4 (119372245-119372407 _163bp)	MERWGKGLHGLEERGDSVPIPKHRAGRRGGVGKRGVRGRELGGQGKYGAGCSECGTRRIAARGEAEFEKAAEEVRHLKTKPSDEEMLFIYGHYKQATVGDINTERPGMLDFTGKAKWDAWNELKGTSKEDAMKAYINKVEELKKKYGI (148 aa)
6 (ACBP-1e)	_	_	_	_	_	P07108-6	_	MSQAEFEKAAEEVRHLKTKPSDEEMLFIYGHYKQATVGDINTGMQSGGWKGICSSKQAQQLRLEVPGNFTLKLPEALLFRWGMVMVPEVEKTMFRILSVSSSNRIQILVLEGLYWPSPAATLY (123 aa)
7	DBI-210	ENST00000542275.5	757	ENSP00000440698.2	_	A0A0A0MTI5	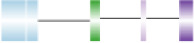 ENSE00003720718/ENSE00002257304 ENSE00003674746/ENSE00003592668 ENSE00001852476 1 (119367048-119367334 _287bp) 2 (119367338-119367463 _126bp) 3 (119368188-119368305 _118bp) 4 (119370740-119370802 _63bp) 5 (119372245-119372407 _163bp)	MGWTSLCSGRGVGVEGAWRDGGRGCTDWRSEETQSPSRSTGQDVAAEWGSEESVAESLEAEFEKAAEEVRHLKTKPSDEEMLFIYGHYKQATVGDINTERPGMLDFTGKAKWDAWNELKGTSKEDAMKAYINKVEELKKKYGI (143 aa)
8	DBI-208	ENST00000535617.5	654	ENSP00000442917.2	CCDS74568	B8ZWD1	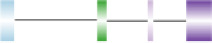 ENSE00002282645/ENSE00003674746 ENSE00003592668/ENSE00001931282 1 (119366924-119367090 _167bp) 2 (119368188-119368305 _118bp) 3 (119370740-119370802 _63bp) 4 (119372245-119372550 _306bp)	MSQVQRVHSQAAKAEFEKAAEEVRHLKTKPSDEEMLFIYGHYKQATVGDINTERPGMLDFTGKAKWDAWNELKGTSKEDAMKAYINKVEELKKKYGI (97 aa)
_	DBI-205	ENST00000460901.1	774	_	_	_	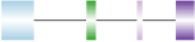 ENSE00001937885/ENSE00003658460 ENSE00003634772/ENSE00001843688 1 (119367081-119367463 _383bp) 2 (119368188-119368305 _118bp) 3 (119370740-119370802 _63bp) 4 (119372245-119372454 _210bp)	No protein
_	DBI-206	ENST00000475783.1	842	_	_	_	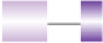 ENSE00001895774/ENSE00001891815 1 (119370217-119370802 _586bp) 2 (119372245-119372500 _256bp)	No protein
_	DBI-207	ENST00000492375.1	784	_	_	_	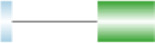 ENSE00001814896/ENSE00001958680 1 (119366935-119367060_126bp) 2 (119368188-119368845 _658bp)	No protein

Note: Isoforms 1-6: 6 described isoforms; Isoforms 7-8: 2 potential isoforms that are computationally mapped. The information are obtained from Uniprot dataset (https://www.uniprot.org/) and e!Ensembl dataset (https://www.ensembl.org/index.html). Gene/transcripts that do not contain an open reading frame or transcripts believed to contain intronic sequences relative to other coding transcripts of the same gene were considered unlikely to code for ACBP isoforms.
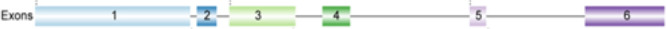

## INCREASED ACBP/DBI PLASMA LEVELS UPON FASTING

The ACBP/DBI orthologue from unicellular fungi (such as *Cryptococcus neoformans, Pichia pastoris* and *Saccharomyces cerevisiae*), filamentous fungi (such as *Aspergillus oryzae)* and facultatively multicellular slime molds (*Dictyostelium discoideum*) was found to be released upon nutrient depletion in an autophagy-dependent fashion [12–16]. Later, it was shown that mouse astrocytes release ACBP/DBI in a way that requires the autophagy machinery to be effective [17]. A similar autophagy-dependent release mechanism for ACBP/DBI was documented for primary human or mouse cells, including circulating leukocytes and hepatocytes cultured under nutrient-free conditions, knowing that nutrient deprivation is one of the most important physiological stimuli of autophagy [7]. Indeed, this starvation-dependent release of ACBP/DBI concerns a pre-existing pool of the protein and is not coupled to an increased transcription of the *ACBP/DBI* gene [7]. In mice, starvation for 24 or 48 hours (which causes, respectively, 10 or 20% weight loss, along with the induction of autophagy) caused a reduction in the abundance of intracellular ACBP/DBI in various organs (heart, kidney, liver, muscle) and a surge in ACBP/DBI plasma levels. This redistribution of ACBP/DBI was inhibited in *Atg4b* knockout mice (which exhibit a partial autophagy defect) or by administration of two pharmacological inhibitors of autophagy, dimethyl α-ketoglutarate and leupeptin [7]. In contrast, the levels of mRNA coding for ACBP/DBI tended to decrease upon starvation in the liver and white adipose tissue from mice [7]. These results suggest that the starvation-induced redistribution of pre-synthesized ACBP/DBI protein depends on autophagy. Although ACBP/DBI is known to be secreted through an unconventional secretory pathway [7], the precise route for its autophagy-dependent release remains to be determined. In humans, voluntary fasting over one to three weeks leads to an increase in plasma ACBP/DBI concentrations [21], while dietary weight loss or weight loss induced by bariatric surgery was coupled to a decrease in ACBPDBI mRNA levels in periumbilical fat [7]. In sum, enhanced translocation of ACBP/DBI protein from the intracellular to the extracellular space, rather than exacerbated ACBP/DBI biosynthesis, explains the fasting-related augmentation of circulating ACPB/DBI protein.

## INCREASED ACBP/DBI TRANSCRIPTION IN OBESITY

Mice that become obese due to a high-fat diet or due to overconsumption of normal chow (due to the leptin deficiency found in animals with the *Ob/Ob* genotype) express high levels of ACBP/DBI mRNA in their livers and white adipose tissue [18]. Similarly, obese insulin-resistant Zucker rats exhibit abnormally high ACBP/DBI levels in their skeleton muscles [19]. Weight gain in mice correlated with enhanced circulating ACBP/DBI concentrations. Since obesity is coupled to an inhibition of autophagy [20–22], it appears improbable that this effect may be explained by an enhanced autophagy-dependent release of ACBP/DBI protein from the intracellular space. Rather, an autophagy-independent release mechanism must account for this observation. Of note, in obese humans, an increase in plasma ACBP/DBI levels was observed as well. Thus, in four distinct independent cohorts, ACBP/DBI concentrations significantly correlated with body mass index (BMI), contrasting with reduced circulating ACBP/DBI levels in patients with anorexia nervosa [7, 23–25]. Moreover, circulating leukocytes from obese individuals contain more ACBP/DBI mRNA than white blood cells from lean subjects [26], and long-term dietary interventions reduced ACBP/DBI mRNA in subcutaneous fat from obese female patients (600 kcal/d for ten weeks) [27], as well as in visceral fat from non-diabetic men or women with overweight or obesity (800-1000 kcal/d for eight weeks) [28]. A similar reduction in abdominal fat ACBP/DBI mRNA was correlated with weight loss in a randomized dietary trial involving a twelve-week-long caloric restriction [29].

In sum, the available evidence suggests that obesity is linked to enhanced transcription of the ACBP/DBI gene, resulting in elevated ACBP/DBI levels.

## ACBP/DBI IN THE PATHOGENESIS OF OBESITY

**Figure 3:**
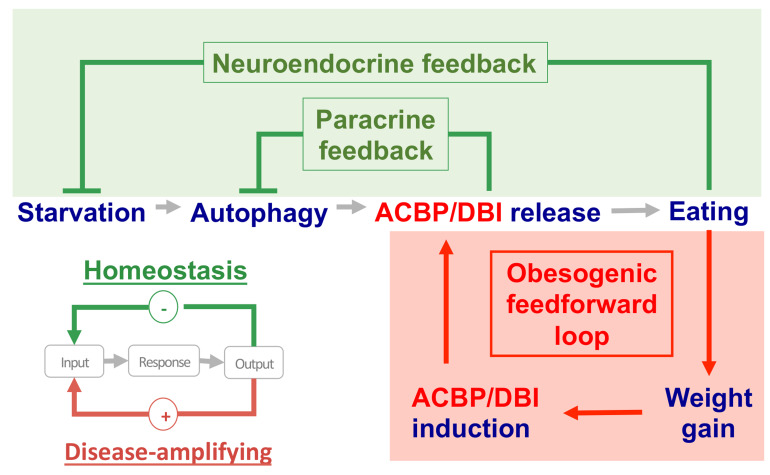
FIGURE 3: Hypothetical involvement of the appetite-stimulatory effects of ACBP/DBI-in two distinct circuitries: (i) an acute neuroendocrine feedback loop that is activated by starvation or fasting and involves the release of pre-formed ACBP/DBI protein from the intracellular to the extracellular space by a mechanism that involves autophagy and (ii) a chronic feedforward loop in which obesity-associate weight gain causes an increase in ACBP/DBI protein levels, likely through transcriptional activation of the *ACBP/DBI* gene. While the feedback loop would reflect homeostatic regulation for the maintenance of energy balance, the feedforward loop would participate in the pathogenesis of obesity.

Starved mice usually exhibit a hyperphagic response when they are allowed to access food pellets. This hyperphagic response can be blocked by injecting a neutralizing antibody against ACBP/DBI into the peritoneal cavity. In contrast, intravenous or intraperitoneal administration of recombinant ACBP/DBI protein (isoform 1) to fed mice inhibits autophagy and was sufficient to induce a rapid hyperphagic response [7, 30]. This latter effect involves GABA receptors of the A type (GABAAR) because mice bearing a point mutation (F77I) in the GABAAR 2 subunit that reduces ACBP/DBI binding [31] fail to increase food intake after ACBP/DBI injection [24]. Thus, the starvation-induced surge in plasma ACBP/DBI may be part of a “hunger reflex” assuring the maintenance of energy and body mass homeostasis [32, 33]. As true for most if not all homeostatic circuitries, this “hunger reflex” would involve a negative feedback loop in which extracellular ACBP/DBI acting on GABAAR would be embedded (**Fig. 3**). In this scenario, starvation-induced autophagy would lead to a surge in extracellular ACBP/DBI, which then acts on GABAAR to stimulate food intake. Once energy and body mass homeostasis are ensured, cessation of autophagy and degradation of circulating ACBP/DBI would cause ACBP/DBI concentrations to return to the basal level, hence closing the homeostatic circuitry.

Contrasting with physiological regulation based on feedback loops, pathologies are often characterized by self-amplifying (“vicious”) feedforward circuitries [34]. As discussed above, obesity is associated with enhanced ACBP/DBI mRNA expression in various organs (e.g. subcutaneous and visceral fat, peripheral blood), as well as with increased levels of circulating ACBP/DBI protein. Thus, the scenario emerges that, in obesity, ACBP/DBI plasma concentrations are constitutively elevated, maintaining a high level of caloric intake, hence perpetuating ACBP upregulation (**Fig 3**). It is tempting to speculate that this pathogenic feedforward circuitry is activated on a different, chronic time scale (which involves transcriptional mechanisms), differing from the physiological feedback regulation, which occurs in an acute, reflex-like fashion (mostly via non-transcriptional mechanisms, such as an autophagy-associated cellular release mechanism). Obesity-induced activation of PPARγ could play a role in the upregulation of *ACBP/DBI,* as members of the PPAR and SREBP families have been shown to enhance transcription of the *ACBP/DBI* gene above baseline levels [35]. Nonetheless, the exact mechanisms accounting for the pathogenic upregulation of ACBP/DBI in obesity remain enigmatic. In particular, the transcription factor(s) activating the *ACBP/DBI* gene, as well as a possible epigenetic regulation, remain to be identified. Moreover, the mechanisms accounting for the passive leakage or active secretion of ACBP/DBI from adipocytes and other cell types are elusive. We anticipate that the elucidation of these pending questions will yield important insights into the pathogenesis of eating and weight disorders.

## Abbreviations:

ACBP: – acyl coenzyme A binding protein,
DBI: – diazepam binding inhibitor,
GABAAR: – γ-aminobutyric acid A receptor.
